# Short and long-term inequity in outpatient medical use by the type of medical institutions in Korea

**DOI:** 10.1093/eurpub/ckac131.109

**Published:** 2022-10-25

**Authors:** JY Yang, JH Lee

**Affiliations:** 1 Transdisciplinary Major in Learning Health Systems, Korea University, Seoul, South Korea; 2 Department of Health Policy and Management, Korea University, Seoul, South Korea

## Abstract

**Background:**

Many countries agree with the horizontal equity that medical resources should be allocated according to medical needs, regardless of income. Although the short-term equity index calculated through cross-sectional data doesn't reflect the dynamics of individual income and medical use, it can be supplemented by the long-term equity index using panel data. Koreans tend to choose expensive but highly specialized services without considering their medical needs because they are free to choose service providers. This study aims to empirically examine how the patterns of outpatient medical use that are not based on medical needs differ in terms of short- and long-term equity for each type of medical institution.

**Methods:**

Using Korea Health Panel Survey(2014-2018), the equity of outpatient medical use(number of visits, medical expenses) of 10,244 people was measured by type of medical institution (tertiary general hospital, general hospital, hospital, clinic, and dentist). Wagstaff&van Doorslaer(2000)’s tool and Jones & Lopez-Nicolas(2004)’s tool were used to calculate the short and long-term horizontal equity index(HI), and mobility index(MI) to compare short and long-term inequity.

**Results:**

In tertiary general hospitals and dentists, there were short and long-term pro-rich inequalities(HI > 0, p < 0.05). As a result of comparison, long-term inequality was greater in the number of visits (MI < 0), while inequality was easing in the long-term in medical expenses(MI > 0) in tertiary general hospitals. In dentists, long-term inequality was less than short-term inequality in both the number of visits and medical expenses (MI > 0).

**Conclusions:**

The short-term equity index is likely to underestimate or overestimate inequity in our society, so a long-term perspective is needed. Inequality patterns for each type of medical institution should be considered in healthcare reforms for fair distribution of medical resources.

**Key messages:**

• Short-term equity index differs from the long-term equity index in outpatient medical use.

• The pattern of short and long-term equity indices may differ by type of medical institutions.

